# Abundant human anti-Galα3Gal antibodies display broad pathogen reactivity

**DOI:** 10.1038/s41598-020-61632-9

**Published:** 2020-03-12

**Authors:** Jens Magnus Bernth Jensen, Mikkel Steen Petersen, Svend Ellerman-Eriksen, Bjarne Kuno Møller, Jens Christian Jensenius, Uffe B. Skov Sørensen, Steffen Thiel

**Affiliations:** 1 Department of Clinical Immunology, Aarhus University Hospital, Aarhus, Denmark; 2 Department of Clinical Microbiology, Aarhus University Hospital, Aarhus, Denmark; 3 Department of Biomedicine, Aarhus University, Aarhus, Denmark

**Keywords:** Antimicrobial responses, Bacterial infection

## Abstract

Antibodies of the IgG class to terminal Galα3Gal (IgG anti-αGal) is abundant in human plasma and are reported to bind most sepsis-causing Gram-negative bacteria. However, these seminal findings, made more than two decades ago, have not been reexamined. Our aim was to assess IgG anti-αGal´s pathogen reactivity. We affinity purified IgG anti-αGal from a therapeutic grade normal human IgG pool applying two rounds of positive selection with Galα3Gal-coupled beads and included removal of column matrix reactive antibodies. The purified antibodies were rigorously characterized in terms of specificity and purity in various solid-phase immunoassays. We used flow cytometry to study reactivity against 100 consecutive clinical isolates diagnosed as cause of sepsis in humans. We found that the purified IgG anti-αGal displays high specificity for Galα3Gal. Also, IgG anti-αGal at 5 mg/L bound 56 out of 100 pathogens with predilection for Gram-positive bacteria binding 39 out of 52 strains. We confirm that although IgG anti-αGal comprise a small fraction of the human antibody pool (~0.1%), these antibodies targets an impressively large part of pathogens causing invasive disease.

## Introduction

The increasing frequency of pathogens resistant to antibiotics poses immense global challenges^1^ and novel therapeutic strategies are crucial. Over millions of years, human ancestors have evolved numerous countermeasures to pathogen threats. Our hard-earned defense mechanisms are obvious assets to build on in development of effective therapeutics to combat pathogens. Intriguingly, one group of human antibodies, IgG antibodies against the carbohydrate structure terminal Galα3Gal (IgG anti-αGal), were more than twenty years ago reported to bind most of sepsis-causing Gram-negative bacterial pathogens, including *Escherichia coli* and species of *Klebsiella*, *Enterobacter*, *Serratia*, *Citrobacter*, *Proteus*, and *Pseudomonas*^2,3^. This broad pathogen reactivity is of interest in the context of developing new therapeutic measures.

IgG anti-αGal were originally reported to comprise 1% of plasma IgG in all humans^4^, corresponding to around 100 mg/L. According to later studies, the average concentration seems to be maybe 10-fold lower^5–7^ and to vary considerably between individuals (>400-fold^7^). IgG anti-αGal is mainly IgG subclass 2 (IgG2)^5^ in accordance with the general finding for anti-carbohydrate antibodies^8–10^. Human plasma also contains anti-αGal antibodies of immunoglobulin classes A and M^11–13^. It is unclear why humans produce antibodies to Galα3Gal. The antigen, Galα3Gal, is not synthesized by simians of the Catarrhini subdivision (which includes humans, other apes, and old-world monkeys) because the gene encoding the essential α1,3-galactosyltransferase was silenced in our common ancestor^14^ whereas all other mammals synthesize Galα3Gal. As Galα3Gal is non-self, Catarrhini are allowed production of IgG anti-αGal which is found naturally occurring in these animals^15^. The prevailing theory is that Galα3Gal-containing antigens presented to our immune system by enteric bacteria ongoing stimulate IgG anti-αGal production^16^.

Despite the abundance of IgG anti-αGal in human plasma, there is a surprising paucity of studies readdressing the reported broad-spectrum pathogen reactivity and its functional role in combatting bacterial pathogens. Clarifications seems desirable in the context of developing novel therapeutics to combat the increasing pathogen threat.

Our aim was to reexamine IgG anti-αGal for reactivity to pathogens. We rigorously purified and characterized the IgG anti-αGal fraction from a therapeutic grade normal human IgG pool. We then investigated the purified IgG anti-αGal for reactivity to pathogens represented by a panel of 100 consecutive clinical isolates diagnosed as the cause of sepsis in humans.

## Results

In two previous studies Hamadeh and coworkers prepared IgG anti-αGal from plasma by a single affinity purification step^2,3^. Such a preparation may contain significant amounts of contaminating antibodies. We added an extra purification step (polishing) and prepared IgG anti-αGal from a therapeutic-grade normal human IgG pool (nhIgG) by two rounds of affinity chromatography on beads coated with Galα3Gal disaccharide. Moreover, we removed column-reactive antibodies by passing the preparation over uncoated beads. After the first affinity isolation (intermediate preparation), we recovered 0.6% of loaded IgG, i.e. a result not very different from the reported 1%^4^. However, our additional purification steps removed most of the IgG collected in the intermediary preparation as only 0.1% of the starting material was recovered in the final preparation of IgG anti-αGal. This yield is in accordance with other reports which found the IgG anti-αGal constitute on average 0.1% of the human plasma IgG^5–7^.

To examine whether the final preparation consisted of genuine IgG anti-αGal, we subjected the material to rigorous characterizations before commencing studies of pathogen reactivity.

### IgG subclass composition

**Figure 1:**
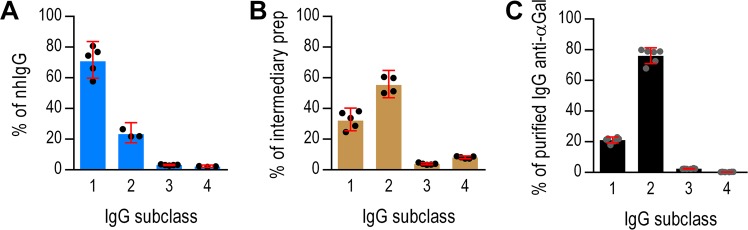
IgG subclass distribution in starting material, intermediate and final preparation of IgG anti-αGal. Distribution of IgG subclasses was determined by a commercial ELISA in the starting material comprised of normal human IgG pool (nhIgG) (**A**), an intermediate preparation acquired after the first affinity isolation (**B**), and in the final preparation of IgG anti-αGal acquired after an additional affinity isolation and removal of column reactive antibodies (**C**). Bars are mean with 95% CI and circles are repeated measurements. Significant differences are identifiable from non-overlapping 95% CIs.

We used ELISA to determine the IgG subclass distributions. The starting material (nhIgG) contained mostly IgG1 (71%), less IgG2 (23%), and some IgG3 (3.2%), and IgG4 (2.2%) (Fig. 1A). In contrast, our intermediate preparation contained more IgG2 (55%) than IgG1 (32%) and more IgG4 (8.1%) than IgG3 (3.9%) (Fig. 1B). This IgG2 proportion is roughly similar to that of the preparation used by Hamadeh *et al*.^2^ according to the characterizations by Yu *et al*.^5^. In our final preparation of IgG anti-αGal, IgG2 constituted 76%; IgG1, 21%; IgG3, 2.4%; and IgG4 only 0.35% (Fig. 1C). This IgG2 proportion is close to that reported for IgG anti-αGal reacting with the Galα3Gal epitope^5^, supporting a high degree of purity of the preparation.

Controls confirmed the specificity of the used ELISA. Only IgG1 was detected when we analyzed a monoclonal IgG1 (rituximab) (Fig. S1A) and a nephelometric assay (which was certified for clinical use) provided an IgG subclass distribution in the starting material similar to our ELISA estimates (Fig. S1B).

In conclusion, we found that the purified IgG anti-αGal was predominantly IgG2.

### Antibody purity

To examine the purity of our final IgG anti-αGal preparation, we determined the recovery of antibodies to different antigens. We examined the reactivity against the disaccharide Galα3Gal, the trisaccharide Galα3Galβ4Glc, and two control antigens: a closely similar trisaccharide (Galα4Galβ4Glc) and an irrelevant protein antigen (tetanus toxoid, TT). Antibodies against TT is present in the starting material (human IgG pool) due to previous vaccinations of the donors. We quantified antibodies by solid-phase immunoassays using antigen coated microtiter wells. These wells are hydrophobic and does not allow direct attachment of hydrophilic carbohydrates. We therefore used glycoconjugates, i.e. di- or trisaccharides coupled to human serum albumin (HSA) for coating. Human plasma contains antibodies against surface-bound HSA that contribute to readings obtained from such glycoconjugates^7^. In accordance, purified IgG anti-αGal did not bind to surface bound HSA whereas nhIgG did so (Fig. S2A). To avoid this problem, we corrected all readings from glycoconjugate-coated wells by subtracting the readings from matching HSA coated wells.

**Figure 2:**
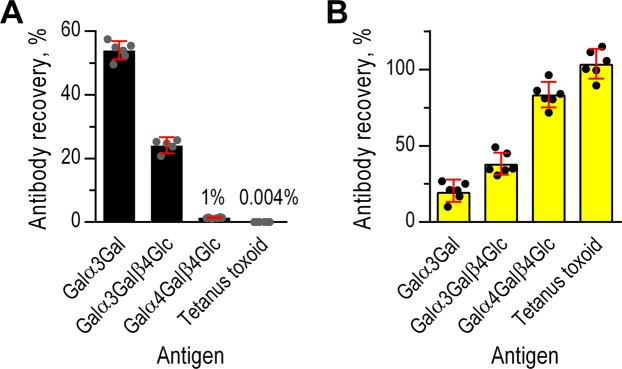
Antibody recovery in the IgG anti-αGal preparation and flow-through. Recovery of different antibodies in the IgG anti-αGal preparation (**A**) and in the flow-through from the affinity column (**B**). Quantifications were made by solid-phase immunoassays (TRIFMA) and the use of standard curves. Bars are mean with 95% CI and circles are repeated measurements. Significant differences are identifiable from non-overlapping 95% CIs.

Recovery of antibody to Galα3Gal (IgG anti-αGal) from the starting material was 54%, recovery of antibody to Galα3Galβ4Glc was 24%, and recovery of antibody to the control antigen Galα4Galβ4Glc were only 1.3% (Fig. 2A). These results demonstrate high selectivity of the used affinity purification. Recovery of antibody to TT in the final preparation was less than 0.005%, signifying effective removal of contaminating antibodies. We used the recovery data to evaluate the fold increase in the ratio of IgG anti-αGal to contaminating antibodies (represented by anti-TT antibodies) in the final IgG anti-αGal preparation compared to the starting material. This unitless factor, *F*, equaled 12,000 (95% CI: 11,000–14,000), meaning that the affinity procedures had increased the content of IgG anti-αGal relative to other antibodies by this factor.

As nearly half of IgG anti-αGal was lost, we found it important to address where in the procedure this occurred. Therefore, we also determined antibody recoveries in the material passing through the column (flow-through) and in the intermediate preparation. As expected, practically all antibody to TT and the far majority of antibody to Galα4Galβ4Glc was present in the flow-through (Fig. 2B). The flow-through also contained considerable parts of antibody to Galα3Gal (19%) and Galα3Galβ4Glc (37%). Of note, IgG anti-αGal in the final preparation and in the flow-through did not add up to 100% (Fig. S2B), i.e. ~27% of antibody to Galα3Gal was lost between the first positive selection and the final elution. In agreement, antibody recoveries in the intermediate preparation were significant higher than those in the final preparation (Fig. S2C). The cumulative recovery of antibody to Galα3Gal in the intermediate preparation and the flow-through actually added up to more than 100% (~130%, Fig. S2D). The surplus may be antibodies of low Galα3Gal avidity (in the flow-through) that were not able to bind to Galα3Gal in the presence of antibodies with higher avidity (during purifications and in the quantitative assay). This issue was not addressed further.

We also determined *F* for the intermediate preparation, which equaled 2,300 (95% CI: 2,000–2,800), i.e., the relative purity was more than five times higher in the final preparation. In conclusion, the extended isolation procedure ensured a substantially improved purity of the final IgG anti-αGal preparation compared to a traditional one-step method. However, we achieved the improved quality at the expense of recovery.

### Properties of purified and native IgG anti-αGal

The stringency of our purification protocol led us to collect only half of the original IgG anti-αGal in the starting material but of high purity. The recovered IgG anti-αGal could possibly differ from IgG anti-αGal in the starting material and we therefore compared selected properties. In these experiments, we used solid-phase immunoassays with the coated antigens Galα3Gal-HSA and HSA (for control) as above.

**Figure 3:**
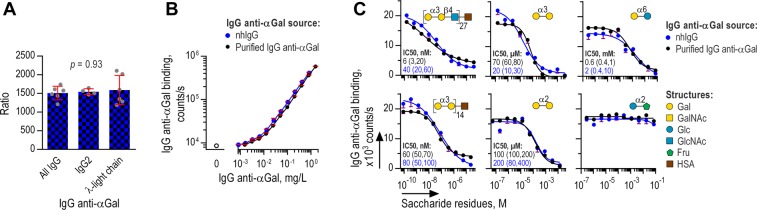
Properties of IgG anti-αGal in the final purified preparation and in the starting material All panels show TRIFMA of antibody binding to solid-phase Galα3Gal. (**A**) Concentration of IgG anti-αGal in purified IgG anti-αGal relative to the starting material (nhIgG) at equimolar total IgG concentration. The ratio was determined for the following subtypes of IgG anti-αGal: All IgG subclasses, IgG2 subclass, and λ-light chain bearing IgG. Bars are means with 95% CI and circles depict repeated tests. Relative concentrations of the three types of IgG anti-αGal were tested for difference by ANOVA (logarithmic transformed data). No difference was evident suggesting that the composition of IgG2 and λ-light chain bearing antibodies among IgG anti-αGal was similar in purified IgG anti-αGal and in the starting material. (**B**) Antibody binding as a function of IgG anti-αGal concentration. Mean and SD of three independent experiments, each analyzed as duplicates. (**C**) TRIFMA of IgG anti-αGal binding to solid-phase αGal challenged by each of six different compounds. Inhibitors´ structures and IC50 (with 95% confidence intervals in parenthesis) are displayed with each set of curves. Mean and SD of duplicates. Each curve is representative of minimum two independent experiments.

First, we addressed potential differences in heavy- and light-chain composition. We limited these investigations to quantification of IgG subclass 2 and IgG carrying λ-light-chain. IgG anti-αGal of these characteristics were quantified using relevant secondary antibodies and the results compared with the content in the starting material. To facilitate evaluations, we performed similar analyses for total IgG anti-αGal (signal obtained with secondary antibody against all human IgG subclasses). Thus, three sets of ratios were generated for the two IgG anti-αGal sources: Ratios of all IgG anti-αGal, ratios of IgG anti-αGal of the IgG2 subclass, and ratios of IgG anti-αGal carrying λ-light-chains. Difference between these three ratio sets would imply that the characteristics differ between the IgG anti-αGal in the two sources. However, no significant differences was observed (ANOVA, *p* = 0.93) (Fig. 3A).

Second, we compared avidity for Galα3Gal from binding curves representing antigen-bound antibody as a function of IgG anti-αGal concentration for the two antibody preparations. Different avidities would reveal different binding curves. However, we found that the curves for the starting material and the final preparation were essentially coincident (Fig. 3B), indicating no difference in avidity.

Third, we compared specificities for different structures by inhibition of IgG anti-αGal binding to solid-phase Galα3Gal. We used six different inhibitors: Glcα2Fru, Galα6Glc, Galα2Gal, Galα3Gal, Galα3Gal-HSA, and Galα3Galβ4Glc-HSA. We found almost identical inhibition curves for both IgG anti-αGal sources (Fig. 3C). Also, the hierarchy of compounds ordered according to half-maximal inhibitory concentration was identical (Spearman’s rho = 1.0, *p* < 0.0001), yielding IC50 for Galα3Galβ4Glc-HSA ~20 nM, for Galα3Gal-HSA ~70 nM, for Galα3Gal ~50 µM, for Galα2Gal ~200 µM, and for Galα6Glc ~10 mM. Glcα2Fru did not inhibit binding in the examined concentration range. Of interest, IgG anti-αGal seems to bind patterns of multiple Galα3Gal-residues markedly better than single Galα3Gal inasmuch as glycoconjugates presenting multiple Galα3Gal residues (on average 14 per HSA molecule, according to the manufacturer) caused markedly more efficient inhibition than the comparable concentration of free Galα3Gal residues (IC50 was approximately 700-fold lower for the glycoconjugate).

In conclusion, we observed no differences between the properties of IgG anti-αGal in the starting material and in the purified IgG anti-αGal which strongly supports that the purified IgG anti-αGal was equivalent to the native IgG anti-αGal.

### Binding of IgG anti-αGal to red blood cells

**Figure 4:**
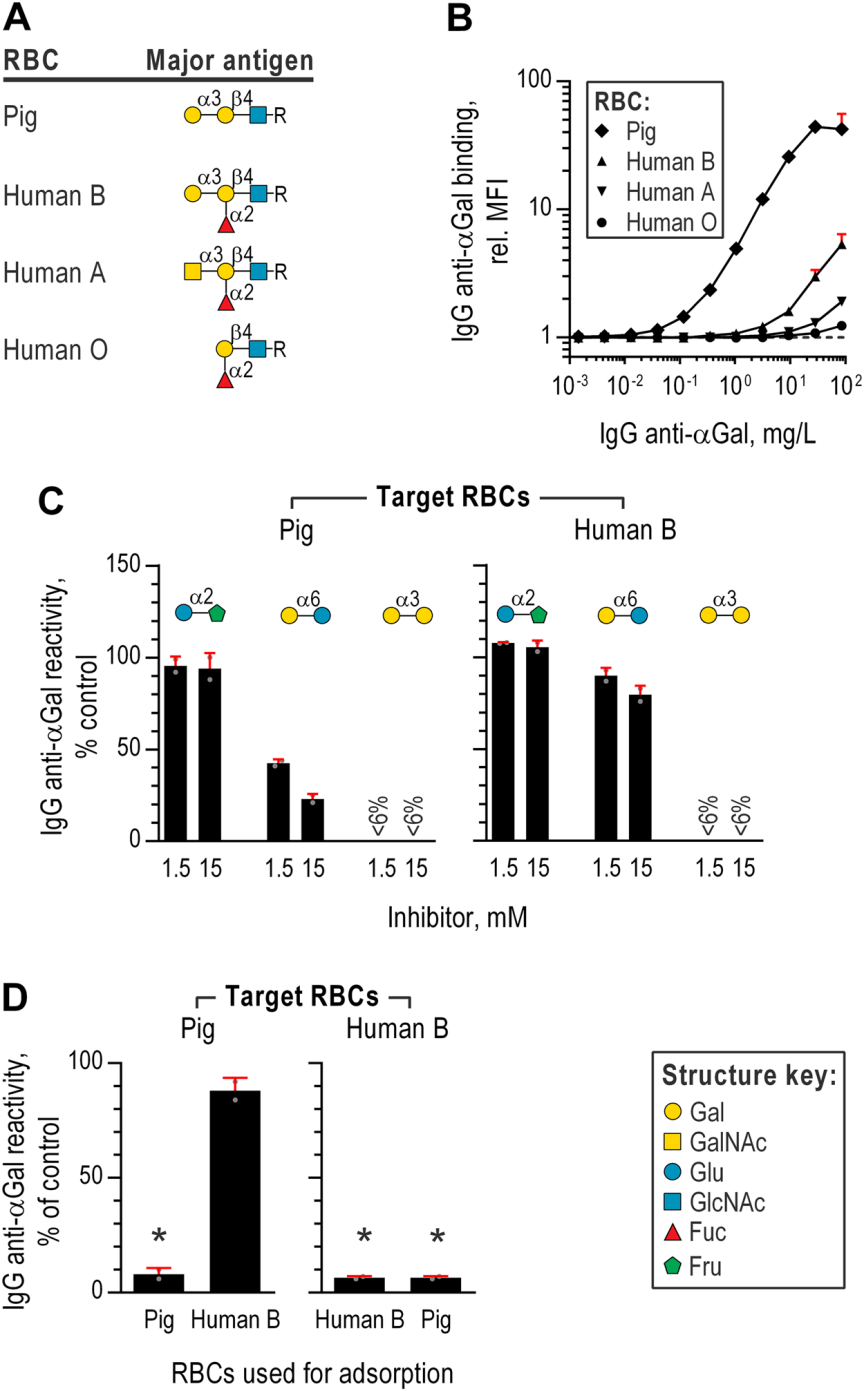
Binding of IgG anti-αGal to red blood cells. (**A**) Symbolic depictions of the major red blood cell (RBC) carbohydrate antigens. Structure key in the lower right. Data in panels (B–D) were acquired by flow cytometry using fluorescent-coupled secondary F(ab’)_2_ against human IgG to detect RBC bound purified IgG anti-αGal. (**B**) IgG anti-αGal reactivity with RBC presenting different major surface antigens (depicted in previous panel). Mean and SD of minimum two independent experiments. (**C**) Reactivity of purified IgG anti-αGal at 5 mg/L with RBCs in the presence of soluble disaccharides (inhibitor) relative to no inhibition (control). Reactivity was calculated from relative MFIs by means of concomitant analyzed standard curves. Mean and SD of two independent experiments. (**D**) RBC reactivity of purified IgG anti-αGal after adsorption on RBCs (pig or human B). Mean and SD of two independent experiments.

We used flow cytometry to study binding of IgG anti-αGal to glutaraldehyde fixed red blood cells (RBCs) from pigs and humans. Bound IgG anti-αGal was detected using fluorescent-coupled F(ab’)_2_ anti-human IgG. Pig RBC cells present Galα3Gal epitopes on the surface at approximately 6 × 10^4^ residues per cell^15^. Apparently, our purified IgG anti-αGal display higher avidity for pig RBCs than the IgG against pig RBC found in normal human serum (Fig. S3). As controls, we included human RBCs, which possess ABO-blood group antigens H, A, and/or B of close structural resemblance to Galα3Gal (Fig. 4A). The ABO-blood group antigens are presented at considerably higher numbers per cell (5 × 10^5^ epitopes per RBC^17^) than Galα3Gal on pig RBC. We expected binding to human RBCs presenting B antigen because persons devoid of B antigen produce IgG anti-αGal cross-reactive with B antigen^18^ and our purified IgG anti-αGal originated from an IgG pool from a large number of humans displaying different ABO-types and most humans lacks B antigen^19,20^. The 10-fold higher number of epitopes per human RBC as compared to pig RBCs likely increase the sensitivity of the assay in detecting IgG anti-αGal bound on B positive human RBCs. Indeed, we observed significant binding to human RBCs, in particular the B antigen positive RBCs (Fig. 4B). Binding-signals equivalent to maximal observed for human RBCs were however achieved at far lower IgG anti-αGal concentrations with pig RBCs (90-fold less for B presenting, 400-fold less for A presenting, and 2,000-fold less for RBCs only presenting H-antigen (i.e., blood group O RBCs)). We had expected a higher level of binding to human B RBCs why we tested RBC reactivity for eight consecutive type B blood donors but found that all produced very similar low-level reactivity (relative MFI ranging between 1.5 and 1.9 with IgG anti-αGal at 5 mg/L). These findings, demonstrate that IgG anti-αGal markedly better binds Galα3Gal than very similar structures presented at higher density.

To test specificity, we challenged the reactivity to pig RBCs and human type B RBCs by each of three inhibitors: Glcα2Fru, Galα6Glc, and Galα3Gal. Residual RBC reactivity was quantified by means of standard curves. For both types of target RBCs, reactivity was abolished completely by Galα3Gal whereas Galα6Glc reduced reactivity partially and Glcα2Fru did not reduce reactivity (Fig. 4C). Of interest, Galα6Glc displayed less inhibition for type B RBCs than for pig RBCs. This probably reflects the larger antigen density on human RBCs allowing comparatively more antibody binding for an equivalent inhibitor concentration. These experiments established specificity of the purified IgG anti-αGal for Galα3Gal on cells.

We addressed the mechanism of IgG anti-αGal´s subtle reactivity with B antigen. Possibly, reactivity could be attributable to a smaller subset of antibodies displaying particular specificity for B antigen or the main part of the antibodies displaying low avidity for B antigen. To clarify, we examined the level of reactivity to pig RBCs after removal of reactive antibodies by adsorption with either human B RBCs or pig RBCs. As expected, adsorption with either type of RBCs abrogated reactivity to human B RBCs and adsorption with pig RBCs abrogated reactivity to pig RBCs (Fig. 4D), proving that adsorption had been effective. However, adsorption with human B RBCs only reduced pig RBC reactivity by around 10%. This suggests that only a small proportion of IgG anti-αGal displayed specificity for B antigen and signifies that IgG anti-αGal are heterogeneous in terms of specificity.

### Binding of IgG anti-αGal to *Escherichia coli* O86

*E*. *coli* O86 has been used as a model of IgG anti-αGal-reactive microorganism^16,21,22^. The lipopolysaccharide of *E*. *coli* O86 contains a B antigen-like structure^23^ but this does not seem to be the primary target of IgG anti-αGal which is unknown^16^. We used formaldehyde fixed *E*. *coli* O86 to optimize our flow cytometry assay for further studies of IgG anti-αGal reactivity with microorganisms.

**Figure 5:**
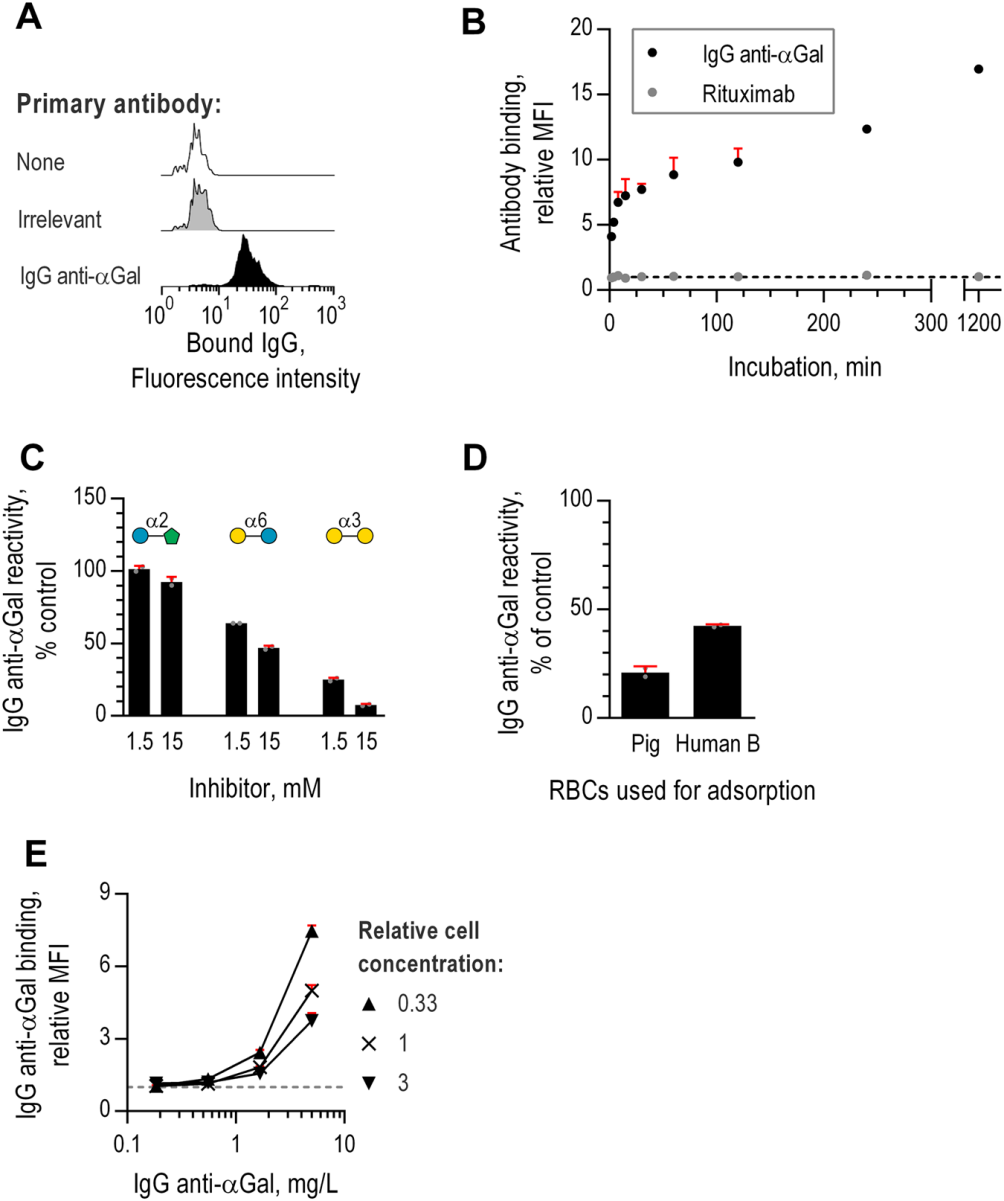
Binding of IgG anti-αGal to *Escherichia coli* O86 examined by flow cytometry. Antibody bound on *Escherichia coli* O86 was detected using fluorescent-coupled F(ab’)_2_ anti-IgG. (**A**) Binding of antibody of irrelevant specificity (anti-CD20, rituximab) at 10 mg/L and IgG anti-αGal at 5 mg/L. (**B**) Bacterial antibody binding as a function of incubation time. Mean and SD of two independent experiments. Black, dotted line: *y* = 1. (**C**) IgG anti-αGal binding as a function of IgG anti-αGal concentration for each of three concentrations of *E. coli* O86. Mean and SD of two independent experiments. Grey, dotted line: *y* = 1. (**D**) Bacterial reactivity of IgG anti-αGal at 5 mg/L in the presence of soluble disaccharides (inhibitor) relative to no inhibition (control). Reactivity was calculated from relative MFIs by means of concomitant analyzed standard curves. Mean and SD of two independent experiments. (**E**) Bacterial reactivity of IgG anti-αGal after adsorption on RBCs (pig or human B). Mean and SD of two independent experiments.

As expected, IgG anti-αGal bound *E*. *coli* O86 (Fig. 5A). Binding signal increased considerably with incubation time from 2 min to 20 hours (Fig. 5B). As expected, velocity of antibody-binding (increase in relative MFI per minute) was highest initially and quickly dropped, reaching essentially zero within 45 min (Fig. S4). Thus, small differences in incubation time (as for setting up parallel experiments) were expectedly far more influenced at short incubation times compared to longer incubation times (>45 min). We therefore settled for an incubation time of 60 min.

To investigate the specificity of IgG anti-αGal binding, we challenged reaction with *E*. *coli* O86 by soluble disaccharide inhibitors (Glcα2Fru, Galα6Glc, and Galα3Gal) in an experimental setup similar to that applied above for RBCs. We found that Glcα2Fru had essentially no effect, Galα6Glc inhibited at most half of reactivity, and Galα3Gal inhibited nearly all reactivity (Fig. 5C), thereby confirming specificity of the binding to *E*. *coli* O86.

To examine whether the B antigen-like structure in the microorganism´s LPS was a target for IgG anti-αGal, we pre-adsorbed our antibody preparation by human B RBCs or pig RBCs. We found that pig RBC adsorption was approximately 2-fold more effective in reducing reactivity with *E*. *coli* O86 (Fig. 5D), which supports the notion that *E*. *coli* O86 presents more effective ligands for IgG anti-αGal than simply a structure mimicking B antigen.

To examine the importance of antibody-to-antigen ratio in our analyses, we incubated serial dilutions of IgG anti-αGal with three different concentrations of *E*. *coli* O86. For each concentration of *E*. *coli* O86, antibody concentration was positive associated to antibody-binding signal and in an exponential manner (Fig. 5E). In accordance, for a fixed IgG anti-αGal concentration, decreasing relative concentration of *E*. *coli* O86 resulted in more than a linear rise in antibody-binding signal. Saturation of antibody-binding was not achieved in these experiments. These results emphasize the obvious that antibody-binding signal is influenced by antibody-to-antigen ratio. In consequence, reporting the level of IgG anti-αGal reactivity for different microorganisms may be of little value unless findings are made under fixed antigen-to-antibody ratio (and similar presentation of antigens). To simplify, we propose reporting reactivity as a categorical variable (positive or negative) and include controls for difference in antigen concentration between groups.

### IgG anti-αGal targets most pathogens diagnosed as cause of sepsis

To provide a broad picture of anti-αGal reactivity with pathogens, we examined binding to 100 consecutive clinical strains, diagnosed as the cause of sepsis in humans. IgG anti-αGal was used at 5 mg/L and thus slightly conservative relative to mean concentration in human plasma of approximately 10 mg/L^5–7^. To correct for possible unspecific immunoglobulin capture by the microorganisms (e.g., by protein A found on some *Staphylococcus aureus*) we tested each pathogen for capture of an irrelevant antibody (rituximab, monoclonal anti-hCD20 at 10 mg/L). We defined cutoff for positive antibody binding as a relative MFI of 1.10, which was the maximal observed binding signal achieved with the irrelevant antibody on microorganisms not possessing IgG capturing proteins (defined from binding studies performed with 91 serotypes of pneumococci, data not shown).

**Figure 6:**
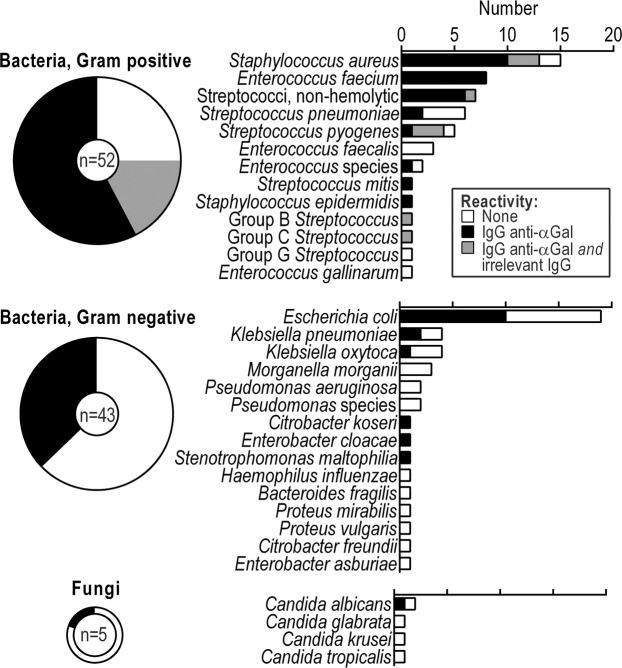
IgG anti-αGal targets most sepsis-causing pathogens. Frequency of antibody reactivity to pathogens. Pathogens were consecutive clinical isolates (*n* = 100) obtained from blood cultures and diagnosed as the cause of sepsis in patients. Reactivity determined by flow cytometry. Each isolate was tested twice. Anti-CD20 was included to reveal binding through bacterial IgG-binding molecules.

The pathogens constituted a diverse collection including Gram-positive and Gram-negative bacteria as well as some fungi and therefore likely present very different surface structures. Nevertheless, we found IgG anti-αGal to bind 56 out of the 100 microorganisms (Fig. 6, representative histograms in Fig. S5A). Of the bacteria, 58% (95% CI: 47–68%) reacted, and one in five of fungi reacted. Among bacteria, 75% (95% CI: 61–86%) of Gram-positive strains reacted, and 37% (95% CI: 23–53%) of Gram-negative strains reacted. Some Gram-positive species reacted particularly frequently: *Enterococcus faecium*, 100% (97.5% CI: 63–100%); non-hemolytic streptococci, 100% (97.5% CI: 59–100%); *Staphylococcus aureus*, 87% (95% CI: 60–98%); and *Streptococcus pyogenes*, 80% (95% CI: 18–99%). Among Gram-negative bacteria, *E*. *coli* reacted particularly frequently at 53% (95% CI: 29–76%). The irrelevant antibody bound some *S*. *aureus* (three out of 15) and some non-pneumococcal streptococci (six out of 17), which we assume possessed IgG-capturing proteins. We did not examine the degree to which unspecific binding accounted for IgG anti-αGal´s binding to these strains. Because of the mentioned inverse relation between IgG anti-αGal reactivity and antigen concentration (Fig. 5E), we compared proxies for microorganisms´ size (forward scatter) and concentration (number of events detected by flow-cytometry) for reactive and non-reactive strains but found no differences (Fig. S5B,C).

In conclusion, highly purified IgG anti-αGal targeted more than half of a broad range of strains from a very heterogeneous collection of pathogens with particular predilection for Gram-positive bacteria.

## Discussion

More than two decades ago, the abundant human antibody, IgG anti-αGal, was reported to bind most of sepsis-causing Gram-negative bacteria^2,3^. Despite the expedience in understanding the human immune response of anti-carbohydrate antibodies, studies reexamining these unexpected observations are lacking. We therefore examined IgG anti-αGal´s reactivity with a collection of 100 consecutive clinical isolates, diagnosed as cause of sepsis in humans, using a rigorously purified preparation of IgG anti-αGal. We found IgG anti-αGal to bind most pathogens, Gram-positive as well as Gram-negative bacteria.

The present study extended and improved the original study by Hamadeh and co-workers^2^ regarding the quality of the used IgG anti-αGal preparations, collection of pathogens, and methods used for investigating reactivity.

The source material in the previous studies was serum or plasma from an undisclosed number of healthy persons^2^, introducing the possibility that the broad-reactivity could be a phenomenon of selected persons. Moreover, additional serum factors, e.g., lectins and IgG anti-αGal of other classes, may be co-purified and influence results. For example, IgM anti-αGal constitutes similar concentrations in plasma as IgG anti-αGal^5,7,24^ and may reportedly block IgG anti-αGal binding^5^. Hamadeh and co-workers claimed that serum anti-Gal was normally exclusively IgG and that this also applied to their preparation^2^. Later, they recognized the existence of IgG anti-αGal of the additional Ig classes in human serum^12^. To achieve a purer system and avoid possible bias introduced by inter-individual differences, we chose a starting material of therapeutic-grade nhIgG prepared from plasma of thousands of donors. Hamadeh *et al*. used the trisaccharide structure Galα3Galβ4GlcNAc^2^ (found on some mammalian tissues) as a ligand for affinity isolation. We chose to use the disaccharide, Galα3Gal, since this is the defining component of the xenoantigen. It is possible that the trisaccharide used by Hamadeh *et al*. allows capture of additional antibodies as some human antibodies may require a larger epitope for proper binding. In accordance, our columns exhibited roughly half as efficient capture of antibody to Galα3Galβ4Glc than of antibody to Galα3Gal (Fig. 2A). We do not think this difference explains the 10-fold greater yield in the original studies. More likely, our more stringent affinity procedure, including two rounds of positive selection interposed by removal of column reactive antibodies, may explain the difference. We found that our additional measures significantly improved purity compared to a single affinity isolation step. Also, we rigorously characterized our preparation and found it in essence to be comprised of genuine IgG anti-αGal, containing only trace amounts of contaminating antibodies. The Hamadeh *et al*. study of pathogen-reactivity did not assess purity of their IgG anti-αGal preparation. Later on, an independent group examined the preparation used by Hamadeh *et al*. and judged it to be impure^5^.

Opposed to the study of Hamadeh *et al*.^2^, we did not focus on Gram-negative bacteria alone. Instead, we examined all kind of microorganisms causing invasive disease, which we believe adds further to the understanding of IgG anti-αGal´s biological importance.

To study reactivity, we used formaldehyde fixed microorganisms and flow cytometry. We expect this approach is more gentle to the presentation of carbohydrate antigens (formaldehyde reacts with amine groups which are absent in most microbial polysaccharides) than the air-drying applied in the previous study^2^ (carbohydrate configuration may differ in aqueous and dehydrated conditions). Importantly, we included an antibody of irrelevant specificity to control for unspecific capture of human IgG by the microorganisms. No such control was included in the original study. We did indeed observe unspecific capture by 9 out of 100 strains (all Gram-positive species). Our finding that none of the Gram-negative bacteria caught the irrelevant antibody may render the lack of this control in the previous study of less concern. Optimally, our irrelevant antibody should have had exactly the same properties as the purified IgG anti-αGal (IgG subclass composition, light-chain composition, glycosylation etc.). However, such an antibody preparation was not available why we selected a monoclonal IgG for control purpose. Moreover, we used our highly purified antibody at 5 mg/L, corresponding to ca. 0.05% of plasma IgG concentration. We conservatively estimate that our preparation is 92% IgG anti-αGal (see Methods). Thus the concentration of any contaminating antibody is reduced by a factor 25,000 (= (0.05% × 8%)^−1^). Concentration of contaminating antibodies would have been 100-times higher if we had used the preparation obtained after the first affinity isolation step at 100 mg/L. The preparation of Hamadeh *et al*. was obtained by a single affinity isolation step using Galα3Galβ4GlcNAc-derivatized beads and elution with Galα6Glc (melibiose). They argued that 100 mg/L of such antibodies was equivalent to the concentration in normal human serum according to an earlier study^4^. The earlier study applied, however, a different isolation protocol (Galα6Glc-derivatized beads and elution with D-Galactose) and the results are therefore not comparable. We find that our more rigorously purified and characterized IgG anti-αGal preparation, used at 5 mg/L, better represents the capability of genuine IgG anti-αGal.

Despite the differences discussed above, we verified frequent reactivity of IgG anti-αGal with Gram-negative bacteria. The frequency of reactivity we observed with Gram-negative bacteria (37%) is however considerably lower than originally reported (66%) (Chi-squared test, *p* = 0.001), which likely is explained by the methodological differences. In addition, we disclosed that Gram-positive bacteria display even more frequent reactivity with IgG anti-αGal than Gram-negative bacteria. This is of interest in light of Gram-positive bacteria predominating among sepsis-causing pathogens^25^.

It may surprise that the broad-pathogen reactivity of IgG anti-αGal has not been reexamined before. Perhaps experimental shortcomings and the lack of explanation for why IgG anti-αGal achieve such broad pathogen-reactivity has been a factor. It seems unlikely that terminal Gal3αGal residue is present on around half of all pathogens. For example, *E*. *coli* rarely present even αGalactose-residues (~6% of lipopolysaccharide serotypes^26^), suggesting expression of terminal Gal3αGal to be even rarer. Despite this, IgG anti-αGal bound 10 out of 19 *E*. *coli* in the present study. We hypothesize that IgG anti-αGal targets additional microbial antigens besides Gal3αGal thereby allowing a broader pathogen-reactivity than limited to Gal3αGal-presenting microorganisms. Indeed, some data support that IgG anti-αGal display polyspecificity by binding structures devoid of Galα3Gal such as DNA and selected human proteins^27,28^. Future studies should clarify the mechanism for IgG anti-αGal´s broad pathogen reactivity.

Another issue for clarification is the biological effect of IgG anti-αGal bound on pathogens. Hamadeh *et al*. reported that IgG anti-αGal bound pathogens more frequently than commensal bacteria^2^. This raises the question how reactive pathogens are able to cause bloodstream infections in humans when plasma IgG anti-αGal concentration is suggested as high as 100 mg/L. This is 100-fold higher than the concentration of IgG against pneumococci required for protection against invasive disease^29^). It was suggested that IgG anti-αGal may counterintuitively facilitate bloodstream infections with selected reactive enterobacteria^2^. This conclusion was based on findings with a single *Serratia* strain, which apparently became serum-resistant when bound by IgG anti-αGal^2^. The generalizability was left unexamined as was the issue of the effect of phagocytes although such studies was reported in progress. Considering the importance of the original studies, further enquiries are imperative. These may benefit from applying suitable animal models and live bacteria to encompass bacterial defenses against human IgG such as IgG cleaving enzymes^30^ etc. Also, studies should investigate the association of IgG anti-αGal levels and bacterial infections in humans.

In conclusion, IgG anti-αGal comprising a small, but significant, part of human antibodies (~0.1%) targets an impressively large segment of pathogens. This broad reactivity may have therapeutic utility in the era of failing conventional antimicrobial therapy.

## Material and methods

### IgG anti-αGal isolation by affinity chromatography

TSK75 beads (HW75F, Tosoh Bioscience GmbH, Japan) were activated by divinyl sulfone and subsequent incubated with Galα3Gal (G203, Dextra Laboratories, Reading, UK) or solvent (sham) as previously described^7^. *First positive selection*: Normal human IgG (nhIgG, Beriglobulin, CSL Behring, King of Prussia, PA) was diluted to 30 g/L in TBS/Tw (10 mM TRIS, 140 mM NaCl, 0.1% (w/v) NaN_3_, 0.05% (v/v) Tween-20, pH 7.4) and 15 mL solution was passed through a column containing 2 mL Galα3Gal-coupled beads at ambient temperature. Flow rate was approximately 0.25 mL/min. The beads were rinsed by slow passage of 25 mL TBS/Tween. Retained IgG was then eluted from the beads by slow passage of glycine buffer (0.1 M, pH 2.5) in 400 µL fractions into tubes with 34 µL Tris-HCl buffer (1 M, pH 8.5) to neutralize the pH immediately. The column was cleansed between repeats of the process (reset by passage of 4 mL glycine buffer followed by TBS/Tween until pH was 7.4 in the effluent). Fractions with at least 0.1 g/L IgG, as measured by spectrophotometry (NanoDrop 1000, Thermo Fisher Scientific, Waltham, MA, USA), were pooled (termed IgG anti-αGal intermediate prep). *Negative selection*: The IgG anti-αGal intermediate preparation was diluted with 7 vol TBS/Tween and samples of 10 mL was passed through a column with 2 mL sham-treated beads (flow rate as above) followed by slow passage of 8 mL TBS/Tween. *Second positive selection*: The flow-through from the previous step was collected directly onto a Galα3Gal-column. This was subsequently rinsed by slow passage of 25 mL TBS/Tween before the retained IgG was eluted at low pH as above. The two sequential processes were repeated with the column reset between repetitions. Fractions with at least 0.1 g/L IgG were pooled as the final IgG anti-αGal preparation.

### Normal human serum (NHS)

Blood samples (4 mL) were drawn from ten random blood donors (Department of Clinical Immunology, Aarhus University Hospital, Denmark) into tubes containing clot activator. After 45 min at ambient temperature, tubes were centrifuged (2000 *g*, 5 min), serum collected, pooled, aliquoted, and stored at −80 °C.

### Red blood cells

Venous EDTA stabilized blood was collected from anonymous, voluntary human blood donors attending the blood bank at Aarhus University Hospital, Denmark in accordance with the Danish legislation and from pigs undergoing experimental surgery at Institute of Clinical Medicine, Aarhus University, Denmark. Blood (3 ml) was washed twice by centrifugation (200 g, 5 min) in phosphate-buffered saline (PBS; pH 7.4), re-suspended in 45 mL PBS with 2% glucose (w/v) and 0.25% glutaraldehyde (v/v), and rotated gently end-over-end for 1 h at ambient temperature. Residual aldehyde groups were inactivated by ethanolamine (2.5 mL, 1 M, pH 8, rotation for 15 min). The RBCs were washed twice by centrifugation (200 *g*, 5 min), first in TBS (10 mM TRIS, 140 mM NaCl, 0.1% (w/v) NaN_3_, 0.05% (v/v), pH 7.4) and then in PBS before resuspension in PBS with 0.1% (w/v) human serum albumin (HSA; CSL Behring) and 0.1% sodium azide (w/v). Hematocrit was determined on a Sysmex XT-1800i (Sysmex Corporation, Japan).

### Microbial strains

*Escherichia coli* O86 was from American Type Culture Collection: (ATCC 12701, LGC standards). Strains from 100 consecutive isolates from blood cultures (Department of Clinical Microbiology, Aarhus University Hospital, Denmark) diagnosed as the cause of sepsis in patients. These strains were included when both a biomedical laboratory scientist and a clinical microbiologist (medical doctor) agreed that the identified microorganism caused sepsis in the particular patient based on the specific isolate and clinical information retrieved from medical charts and contact to the clinician treating the patient leading to recommendation of treatment. Microorganisms were cultured in 50 mL Todd-Hewitt broth overnight at 35 °C in an incubator (5% CO_2_). The cells were collected by centrifugation (2000 *g*, 30 min) and re-suspended in PBS with 1% formaldehyde (v/v). The next day, the fixed cells were washed twice in 50 mL PBS before re-suspension in 10 mL TBS to block residual aldehyde groups. The microorganisms were centrifuged and the supernatant discarded before re-suspension of the collected cells in TBS and storage at 4 °C until use.

### Antibody biotinylation

One mg antibody solution was dialyzed two times against PBS (pH 7.4, 2 h and then overnight) and one time against PBS (pH 8.5, 3 h). *N*-hydroxysuccinimidobiotin (Sigma-Aldrich) was added (170 µL of 1 mg/mL in DMSO) and reacted for 4 h before termination by three rounds of dialysis against TBS, pH 7.4 (overnight and twice for 1.5 h).

### Time-resolved immunofluorometric assays (TRIFMA)

The protocol is described in detail elsewhere^7^. Coating compounds were 1) HSA, 2) tetanus toxoid (985 Lf/mL, product number 2674, Statens Serum Institut, Denmark) prediluted 1:249 in RPMI-1640, or 3) glycoconjugates (all from Dextra Laboratories, UK): Galα(1-3)Gal-HSA (NGP2203), Galα(1-3)Galβ1-4Glc-HSA (NGP2330), and Galα(1-4)Galβ1-4Glc-HSA (NGP2340). Antigens were diluted in 0.1 M carbonate buffer (pH 9.4): Glycoconjugates and HSA were at 1 mg/L and tetanus toxoid was diluted 1:39. Microtiter plates (FluoroNunc, Thermo Scientific Nunc A/S, Denmark) were coated overnight in a humid chamber at 4 °C. The wells were emptied and unoccupied binding sites were blocked with TBS/Tween/HSA (TBS/Tween with HSA at 1 g/L). Each of the subsequent steps was separated by three wash cycles in TBS/Tween performed using an automated plate washer. Then, 100 µL samples, diluted in TBS/Tween/HSA with 10 mM EDTA, were applied per well in duplicates and reacted overnight in a humid chamber at 4 °C. The wells were applied 100 µL biotin anti-human IgG (polyclonal rabbit, Dako, Denmark), biotin anti-human lambda light chain (monoclonal murine, 2G9, product ab1943, Abcam, UK), or biotin anti-human IgG2 (monoclonal murine, 3C7, product ab1935, Abcam). All secondary antibodies were used at 1 mg/L in TBS/Tween/HSA and allowed to react for 1 h at ambient temperature. A volume of 100 µL europium-labeled streptavidin (product 1244-360, PerkinElmer), diluted 1:1000 in TBS/Tween with 25 µM EDTA, was applied to each well and reacted for 1 h at ambient temperature. A volume of 200 µL enhancement-solution (PerkinElmer) was finally loaded per well, and the plates were shaken for 5 min before the measurement of time-resolved fluorescence (VICTOR X5 multilabel plate reader, PerkinElmer). Readings from glycoconjugate coated wells were corrected by subtraction of the values of the equivalent readings of HSA coated wells. *Antibody quantification*: Log_10_-transformed data from standard curves were used to approximate formulas (by third-degree polynomials) for concentration as function of TRIFMA reading in Microsoft Excel 2010 or 2016 spreadsheets (Microsoft Corporation, WA, USA). In the text, calculated concentrations are presented on a linear scale. To enable quantification of IgG anti-αGal in mg/L, we related the signals of the standard sample to those obtained from serial dilution of purified IgG anti-αGal at known concentration. The measured concentration of the latter was subtracted 8% because the preparation was assumed 92% pure IgG anti-αGal (see below). *Inhibition experiments:* Compounds used as inhibitors were Galα3Gal disaccharide (Dextra Laboratories), Galα2Gal disaccharide (Dextra Laboratories), melibiose (Galα6Glc, Sigma-Aldrich), sucrose (Glcα2Fru, Merrild Professional ApS, Denmark), Galα3Gal-HSA (Dextra Laboratories), and Galα3Galβ1-4Glc-HSA (Dextra Laboratories). Serial dilutions of the compounds in TBS/Tween/HSA/EDTA were mixed with an equal volume of antibody solution (fixed concentration) and incubated for 1 h at ambient temperature before applied to the wells.

Fold increase (*F*) of IgG anti-αGal to contaminating antibodies in the affinity purified preparation (*prep*) compared to in the starting material (*start*) was calculated from the recoveries (*R* = *N*_*prep*_/*N*_*start*_, *N*_*i*_ = *C*_*i*_ $\times$ *V*_*i*_) of antibody to Galα3Gal (*α*) and tetanus toxoid to represent other antibodies (*U*):

$$F=\frac{{R}_{\alpha }}{{R}_{U}}=\frac{\frac{{C}_{\alpha ,prep}\cdot {V}_{prep}}{{C}_{\alpha ,start}\cdot {V}_{start}}}{\frac{{C}_{U,prep}\cdot {V}_{prep}}{{C}_{U,start}\cdot {V}_{start}}}=\frac{\frac{{C}_{\alpha ,prep}}{{C}_{\alpha ,start}}}{\frac{{C}_{U,prep}}{{C}_{U,start}}}=\frac{{C}_{\alpha ,prep}\cdot {C}_{U,start}}{{C}_{\alpha ,start}\cdot {C}_{U,prep}}$$

*F* was calculated to be 12,000.

The equation can be rearranged to allow estimation of IgG anti-αGal´s relative part of the preparation:

$${C}_{\alpha ,prep}=\frac{F\cdot {C}_{\alpha ,start}\cdot {C}_{U,prep}}{{C}_{U,start}}$$

Contaminating antibodies in the preparation, ${C}_{U,prep}$, constitute $(100 \% -{C}_{\alpha ,prep})$; *F* equaled 12,000;${C}_{\alpha ,start}$ was assumed to constitute 0.1% of the starting material and other antibodies (${C}_{U,start}$) therefore 99.9%. Thus:

$${C}_{\alpha ,prep}=\frac{12000\cdot 0.1 \% \cdot (100 \% -{C}_{\alpha ,prep})}{99.9 \% }\approx 12-12\cdot {C}_{\alpha ,prep}$$

Thus IgG anti-αGal´s percentage of antibodies in the preparation approximate:

$${C}_{\alpha ,prep}=\frac{12}{13}\approx 92 \% $$

Based on similar calculations, IgG anti-αGal´s percentage of antibodies in the intermediate preparation approximate 70%.

### IgG subclass quantification

For quantification of IgG subclasses, a commercial ELISA kit (IVD and CE approved, M1551, Sanquin, the Netherlands) was used according to the manufacturer’s instructions. Calculations were done as described for quantitative TRIFMAs. The distribution of IgG subclasses in the starting material was also determined by turbidimetry (commercial accredited method for routine clinical use, Department of Clinical Biochemistry, Aarhus University Hospital, Denmark) with consistent results.

### Flow cytometry

#### Antibody binding

RBCs and microbial cells were diluted in PBS/HSA (pH 7.4, 1 mg HSA/mL) to obtain a final acquisition rate of 100 events/s for formaldehyde fixed RBCs or 500 events/s for microorganisms. A 10 µL cell suspension was mixed with 10 µL PBS/HSA with or without antibodies. Antibody concentration in the primary incubations were as follows (unless otherwise stated): purified IgG anti-αGal at 5 mg/L or irrelevant antibody (rituximab, monoclonal IgG1, chimeric mouse/human anti-hCD20, Roche, Switzerland) at 10 mg/L. Reactions proceeded for 60 min at 37 °C. Cells were then washed in 1 mL PBS/HSA by centrifugation (2000 *g*, 10 min (microorganisms) or 200 *g* 10 min (RBCs)). Cells were re-suspended in 20 µL PBS/HSA with 1% (v/v) solution of fluorescein-isothiocyanate-coupled polyclonal rabbit F(ab’)_2_ anti-human IgG (F0315, DAKO). The secondary incubation was done in the dark at ambient temperature for 30 min. Samples were diluted with 200 µL filtered (0.45 µm) flow buffer (BD FACSFlow, BD Biosciences, San Jose, CA, USA). Data acquisition was done on a BD FACSCanto Cell analyzer (BD Biosciences). Before each run, the instrument was adjusted using the following strategy. First, forward and side scatter acquisition thresholds were assigned their minimum set points. Second, filtered flow buffer was sampled at medium flow rate while the voltage of forward- and side-scatter detectors were adjusted to obtain an acquisition rate between 0 and 4 events/s. These settings were maintained while samples were subsequently analyzed. Signal height of forward scatter, side scatter, and fluorescein-isothiocyanate fluorescence (excitation 488 nm, emission 530/30 nm) were acquired. Each sample was pre-sampled (5 s) before data was recorded for 20 s. The instrument was rinsed between each sample. To control for bacterial spillover, samples of filtered flow buffer were interposed between each microorganism strain (event rate below 1% of associated samples with microorganisms). Observed final event rates of microorganisms ranged between 170/s and 980/s. Data were analyzed using FlowJo software (version 9.7.6, FlowJo LLC, Ashland, OR, USA) and exported to Microsoft Excel spreadsheets. Rel. MFI: Median fluorescence intensities for a cell incubated with primary antibody relative to parallel incubation in PBS/HSA only. This unitless relative MFI thus approximate one for decreasing densities of bound primary antibody. *Positive antibody reaction*: Mean of two independently determined relative MFIs at least two standard deviations above 1.10. This limit of 1.10 was the maximal observed relative MFI found for the irrelevant antibody reaction with each of 91 pneumococcal serotypes in preliminary experiments. These organisms do not possess IgG-capturing proteins. *Inhibition experiments*: Inhibitor compounds, as listed above, were included together with IgG anti-αGal. Results were compared to experiment without inhibitor. *Adsorption of IgG anti-αGal by RBCs:* IgG anti-αGal, 220 µL at 15.5 mg/L, was mixed with 19 µL suspension of RBCs (human type O, human type B, or pig) at a hematocrit 25%. Reaction was allowed at 37 °C for 30 min before centrifugation (200 *g* 10 min). Supernatant, 220 µL, was mixed with RBCs as above. The procedure was repeated seven times. Had IgG anti-αGal only been lost to dilution, then the concentration would be 10 mg/L in the final supernatant. The final supernatant was passed through a 0.2 µM filter. The final supernatant was mixed with cell suspensions in a 1:1 ratio for cell binding studies. Results obtained for reactivity after human B RBC or pig RBC adsorption were expressed relative to similar results obtained after human O RBCs adsorption. *Quantification of antibody reactivity*: Sampled median fluorescence intensities were converted to measures of antibody reactivity via concurrent standard curves (2-fold dilution series). Data handling was done as described for quantification by TRIFMA.

### Statistics

Frequencies were compared by Pearson’s chi-squared test when all categories contained minimum 10 observations and otherwise the Fisher’s exact test was used. Confidence intervals were calculated using the *t*-distribution (continuous variables, on log_10_ transformed data and then retransformed to linear scale for presentation) or the binomial distribution (dichotomous variables). In general, 95% confidence intervals are reported except for dichotomous variables in the event all observations achieved the same outcome. In the latter case, 97.5% confidence intervals are reported. Tests of relationship between two variables were performed using Spearman correlation unless otherwise stated. We made no corrections for multiple comparisons (to limit risk of type II errors), arguing that the single tests were only exploratory in an overall context. We only performed tests we found directly relevant for the context (to limit risk of type I errors). Data analyses were performed in GraphPad PRISM v. 6.07 (GraphPad Software, CA, USA) and STATA 11 (StataCorp LP, TX, USA). The level of significance was defined to 0.05.

### Ethics

Human material (blood) was collected from excess blood donated by anonymized voluntary blood donors at the blood bank, Department of Clinical Immunology, Aarhus University Hospital, Denmark in accordance with the Danish legislation. All donors had provided informed consent of such use. All methods were carried out in accordance with relevant guidelines and regulations. The studies were approved by *The Danish Data Protection Agency* (reference number 1-16-02-40-12/2007-58-0010) and the *Ethics Committee in Central Denmark Region* (reference number 1-10-72-127-12).

## Supplementary information

Supplementary data file.
