# Supplementary material for: Abundant human anti-Galα3Gal antibodies display broad pathogen reactivity
Source: Sci Rep. 2020 Mar 12;10:4611. doi: 10.1038/s41598-020-61632-9 (PMC7067764; doi:10.1038/s41598-020-61632-9)
Supplement: Supplementary file 1 — Supplementary data file. [file 41598_2020_61632_MOESM1_ESM.docx]

**Abundant human anti-Galα3Gal antibodies display broad pathogen reactivity**

Jens Magnus Bernth Jensen,*^,1^ Mikkel Steen Petersen,^1^ Svend Ellerman-Eriksen,^2^ Bjarne Kuno Møller,^1^ Jens Christian Jensenius,^3^ Uffe B. Skov Sørensen,^3^ and Steffen Thiel.^3^

*Corresponding author, [jejensen@rm.dk](mailto:jejensen@rm.dk)

^1^Department of Clinical Immunology, Aarhus University Hospital, Denmark

^2^Department of Clinical Microbiology, Aarhus University Hospital, Denmark

^3^Department of Biomedicine, Aarhus University, Denmark





Figure S1. **IgG subclass distributions examined by ELISA**

(**A**) Distribution of IgG subclasses in monoclonal IgG1 (rituximab) estimated by ELISA. (**B**) Distribution of IgG subclasses in nhIgG determined by ELISA and clinical grade nephelometric assay. Bars are mean with 95%CI and circles represent repeated measurements. Nephelometry was performed once.





Figure S2. **Antibody reactivity in fractions from affinity isolation**

(**A**) TRIFMA of IgG binding to surface coated antigens. Mean and SD (covered by symbols) of duplicate analyses. (**B**–**D**) Recovery of selected antibodies in antibody preparations. Quantifications were made by solid-phase immunoassays (TRIFMA) and standard curves. Bars are mean with 95%CI and circles are individual measurements. Significant differences are identifiable from non-overlapping 95%CIs.





Figure S3. **Reactivity of purified IgG anti-αGal and antibody in normal human serum against pig RBCs**

Stock solutions of purified IgG anti-αGal (180 mg/L) and normal human serum (NHS) (pool from ten random blood donors) were serial diluted and incubated with fixed pig RBCs. We measured IgG on RBC with fluorescent-coupled anti-hIgG and flow cytometry. Signals were corrected for background by subtracting signals from experiments without primary antibodies. Signal as function of concentration were compared by linear regression (log_10_-transformed data, *R*^2 ≥ 0.99, dotted lines are 95%CIs). The slope associated with purified IgG anti-αGal was significantly higher than that associated with NHS, meaning that more IgG are bound for an equal increase in IgG concentration. This strongly support higher avidity of the anti-pig RBC antibodies in purified IgG anti-αGal compared to those in NHS.





Figure S4. **Velocity of IgG anti-αGal binding to *Escherichia coli* O86**

Velocity of IgG anti-αGal binding was determined using GraphPad software as the first derivative of data presented in **Figure 5B**.





Figure S5. **IgG anti-αGal pathogen reactivity**

(**A**) Different distributions of IgG anti-αGal binding to various bacterial strains. Note binding of anti-CD20 (irrelevant antibody) to isolate #13 in center panel. (**B**) Number of acquired events for IgG anti-αGal reactive and non-reactive strains. Mean with 95%CI. Due to the 95%CI overlap, no difference in means were evident. Assuming event number reflects isolate concentration, this supports that the two groups were examined at similar concentrations. (**C**) Forward scatter for IgG anti-αGal reactive and non-reactive strains. Median with interquartile range. Mann-Whitney U test used for comparison (*p* = 0.20). Assuming forward scatter reflects isolate size, this supports that the two groups displayed similar median size.
